# Managed Bumblebees Outperform Honeybees in Increasing Peach Fruit Set in China: Different Limiting Processes with Different Pollinators

**DOI:** 10.1371/journal.pone.0121143

**Published:** 2015-03-23

**Authors:** Hong Zhang, Jiaxing Huang, Paul H. Williams, Bernard E. Vaissière, Zhiyong Zhou, Qinbao Gai, Jie Dong, Jiandong An

**Affiliations:** 1 Key Laboratory for Insect-Pollinator Biology of the Ministry of Agriculture, Institute of Apicultural Research, Chinese Academy of Agricultural Sciences, Beijing, China; 2 Department of Life Sciences, The Natural History Museum, London, United Kingdom; 3 INRA, Laboratoire Pollinisation & Ecologie des Abeilles, UR406 Abeilles & Environnement, Avignon, France; Monash University, AUSTRALIA

## Abstract

Peach *Prunus persica* (L.) Batsch is self-compatible and largely self-fertile, but under greenhouse conditions pollinators must be introduced to achieve good fruit set and quality. Because little work has been done to assess the effectiveness of different pollinators on peach trees under greenhouse conditions, we studied ‘Okubo’ peach in greenhouse tunnels near Beijing between 2012 and 2014. We measured pollen deposition, pollen-tube growth rates, ovary development, and initial fruit set after the flowers were visited by either of two managed pollinators: bumblebees, *Bombus patagiatus* Nylander, and honeybees, *Apis mellifera* L. The results show that *B*. *patagiatus* is more effective than *A*. *mellifera* as a pollinator of peach in greenhouses because of differences in two processes. First, *B*. *patagiatus* deposits more pollen grains on peach stigmas than *A*. *mellifera*, both during a single visit and during a whole day of open pollination. Second, there are differences in the fertilization performance of the pollen deposited. Half of the flowers visited by *B*. *patagiatus* are fertilized 9–11 days after bee visits, while for flowers visited by *A*. *mellifera*, half are fertilized 13–15 days after bee visits. Consequently, fruit development is also accelerated by bumblebees, showing that the different pollinators have not only different pollination efficiency, but also influence the subsequent time course of fertilization and fruit set. Flowers visited by *B*. *patagiatus* show faster ovary growth and ultimately these flowers produce more fruit. Our work shows that pollinators may influence fruit production beyond the amount of pollen delivered. We show that managed indigenous bumblebees significantly outperform introduced honeybees in increasing peach initial fruit set under greenhouse conditions.

## Introduction

Peach *Prunus persica* (L.) Batsch is a popular and healthy summer fruit in most temperate regions of the world. China, where peach originated, has by far the largest planted area and is the largest peach producer world-wide, with over 12 million tons produced in 2012 [1]. Being highly perishable and climate-limited, peach fruit yield follows a strongly seasonal supply pattern. Protected cultivation of peach trees can help growers overcome some of the limitations, offering a highly profitable inter-seasonal harvest. In north China, protected cultivation of peach trees has reached the point where there are advantages from large-scale cultivation [2], but pollination remains a limiting factor.

Insect pollinators are important in agriculture [3–6] and make a huge economic contribution globally [7, 8]. Although peach is a self-compatible crop [9] and many varieties are considered self-fertile in the open as autonomous self-pollination is usually adequate to achieve commercial fruit set [10, 11]. Yet there exists considerable intraspecific variation in the level of self-fertility due to differences in floral morphology, especially the spatial arrangement of the anthers relative to the stigmatic surface [9]. Indeed, several authors have reported increased fruit set when the flowers are visited by bees [12–15]. In the absence of wind and insects, as in greenhouses, commercially acceptable levels of fruit set and good fruit quality are difficult to achieve, especially since anther dehiscence can be considerably reduced due to the high humidity under the plastic cover of greenhouses [16].

The most commonly used managed pollinator world-wide [5] is the western honeybee, *Apis mellifera* L. and this species was introduced into China a century ago. However, it is often not the most effective pollinator for all crops or under all conditions [17, 18]. The value of bumblebees as pollinators in agriculture has long been recognized, especially for crops grown in greenhouses [19, 20]. One of the best indicators for assessing pollination effectiveness in self-compatible species is the amount of conspecific pollen deposited on the stigma [21, 22]. Compared with honeybees, bumblebees are often better at depositing conspecific self- or cross-compatible pollen for crops, including watermelon *Citrullus lanatus* [23], apple *Malus domestica* [24] and pumpkin *Cucurbita pepo* [25]. However, all of these measurements were made in the open and none were made in greenhouses.

Pollen deposition is known to affect pollen-tube performance within the pistil [26]. Pollen-tube performance is often measured as pollen-tube length in the style, which is a clear predictor of successful pollination and fertilization [27]. Although much work has been done on the pollen-deposition abilities of pollinators, relatively little work has been done on subsequent pollen-tube performance. It is still unknown how these pollinators affect the later stages of the fertilization process and subsequent fruit development.

Our aim is to explore differences between the effectiveness of the introduced honeybee, *A*. *mellifera* L., and a native Chinese bumblebee, *Bombus patagiatus* Nylander, when used as managed pollinators for peach trees grown in greenhouses. We seek to quantify their effects on different stages of the pollination process in order to understand how differences in pollinator performance arise.

## Materials and Methods

### Study site and management of greenhouses

The experiments were carried out from 25 January to 21 March in 2012, 12 February to 29 March in 2013, and from 26 January to 5 March in 2014, in plastic greenhouse tunnels at Pinggu, Beijing, China, situated at 40.19° N 117.17° E, with an elevation of 65 m. The greenhouse tunnels were privately owned by a local farmer Mr Qirui Ju, who gave permission for the study. No protected or endangered species were involved in the study. The climate in Beijing belongs to the warm temperate zone, and is a semi-humid monsoon-influenced continental climate with four clearly distinct seasons. The ground area of each greenhouse tunnel was 525 m^2^ (75 m × 7 m), the height at the eaves was 2.3 m, and the ridge height was 3.2 m. The back wall and the side wall were built of bricks. The sunny side of the greenhouse was covered with two transparent polyethylene film layers (ultraviolet blocking), overlapping each other on top of the greenhouse. One of the layers can be opened and closed in order to regulate the temperature of the greenhouse during the day. A fly net was fixed on the gaps between the two film layers at the top of the greenhouse to prevent bees from flying out when the layer was opened. A heat preservation quilt, controlled by a roll blind machine, was laid on the surface of the plastic layer to reduce heat losses at night.

Peach trees (*Prunus persica* ‘Okubo’) were planted in 2006 in rows 3 m apart and spaced 1.5 m apart within the rows, with a total of 90 trees planted in each greenhouse. Normal horticultural care was applied in the greenhouse tunnel. The greenhouse soil was covered with a plastic film to save water and suppress weeds. Under greenhouse conditions, the peaches are harvested between May and June followed by organic manure application. After the harvest, the greenhouse stay fully open and the peach trees are exposed to the outside environment both day and night. In early autumn, organic manure and small amounts of compound fertilizer are used by basal furrow application followed by watering. Around mid-November, the greenhouse is covered with polyethylene film during the day and fully covered with the heat preservation quilt at night to keep warm. Irrigation is reduced and even suspended after the greenhouse is covered, especially during blooming. The blooming time of Okubo is around February when there is an average temperature of -8°C to +3°C outside the greenhouse, typical of winter weather in Beijing (blooming time might vary among years according to the annual weather conditions). The sun is the only energy source in the greenhouse, so the heat preservation quilt is rolled up at 8:30 hrs during blooming time. The upper part of the plastic film is opened whenever the greenhouse temperature exceeded 25°C and the percentage of film open is set according to the temperature inside. The greenhouse is fully closed and covered with the heat preservation quilt at 16:30 hrs every day. Before the fruit-stone hardening stage, nitrogen manure is added by top application.

### Pollinator treatment

**Fig 1:**
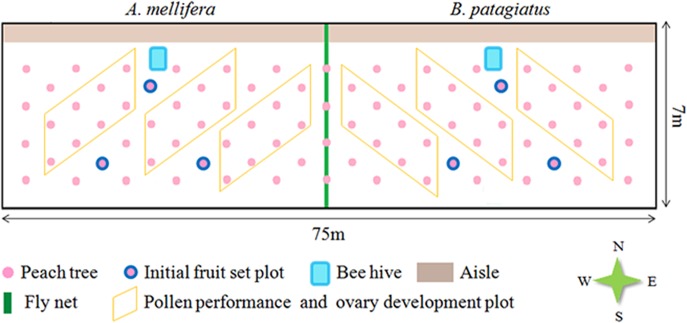
Layout of sampling plots of peach trees in greenhouses.

Greenhouses were separated into two sections by a flight net. When the peach trees were at the early bloom stage, bee hives were placed in the middle of the greenhouse along the aisle in the north (Fig. 1). Both *A*. *mellifera* and *B*. *patagiatus* (the identity of this bumblebee species has recently been revised [28]) were provided from the Institute of Apiculture, Chinese Academy of Agricultural Sciences. At the beginning of the pollination treatment, each *B*. *patagiatus* colony contained 60–70 workers and each *A*. *mellifera* colony contained three frames of about 6000 workers. We placed one hive of either *A*. *mellifera* or *B*. *patagiatus* in each half of the greenhouse. Based on our earlier studies, honeybees start foraging at 14.8±0.4°C (mean ± SD) and forage for 5.9±0.3 h per day, while bumblebees start foraging at lower temperature (8.7±0.2°C) and forage over a longer period of 8.2±0.2 h per day in peach greenhouse[29]. The pollen deposition, pollen-tube performance, and the early development of the ovary were analyzed at the scale of individual flowers. The fruit set was analyzed at the scale of a branch. The layout of the sampling plots is shown in Fig. 1.

### Peach mating system

To investigate the mating system of Okubo peach, the initial fruit set under different pollination treatments was tested. Prior to anthesis, flowers were bagged. After flowers opened, one third of the bagged flowers were hand-pollinated with a soft brush with pollen from the same flower, one third were hand-pollinated with pollen from different flowers, and the remaining flowers remained bagged to exclude pollinators as a control. The hand-pollinated flowers were re-bagged to prevent visits by bees. Bags were left on flowers until the flowers were no longer receptive. Prior to the commercial thinning carried out in the greenhouse (five weeks after full blooming), fruit were counted to measure the initial fruit set.

### Pollen deposition

Pollen deposition on virgin stigmas was tested after a single bee visit, or after many bee visits on the first day of anthesis. Flower buds were selected randomly at the balloon stage (one day before anthesis), usually in the afternoon, and isolated individually in fabric mesh bags (2 mm) to prevent unwanted visits by insects. The flowers in the bags opened normally and did not jostle. Once the flowers had opened the next morning, they were uncovered and monitored by camera until a single bee visit was recorded (for the single-visit deposition test). The stigmas were exposed to bees on the first day of anthesis (for the one-day open pollination test).

Stigmas from each treatment were removed with clean forceps and stored in Eppendorf tubes (1.5 mL). Stigmas were stained with malachite green solution (25 μL malachite green 0.1% aqueous solution dissolved in 10 mL 1% NaCl aqueous solution). Adhering pollen grains were separated from the stigmas in a 100 W ultrasonic bath for one minute (JY92-ⅡDN, China). The pollen grains in the solution were collected by vacuum filtration, using a filter membrane with a pore size of 20 μm (the size of the peach pollen is about 56×30 μm [30]). Once collected, the filter membrane was placed on a slide with a drop of glycerin. A scanner (Nikon Coolscan 9000 ED, Japan) was used to capture digital images of each sample of pollen grains. Image J was used to analyse the images and to count the pollen grains.

### Pollen-tube performance

We compared the pollen-tube performance in flowers pollinated by the two bee species. When sampling in greenhouses, pistils were collected from different part of the greenhouse to detect whether micro-environment had any effects on the fertilization process. For each treatment, a total of 12 pistils at 1, 3, 5, 7, 9, 11, 13, and 15 days after pollination were collected respectively and fixed in FAA solution at 4°C [31]. The fluorescence standard method was used to analyse the pollen performance [32]. Tissues were softened in 4 M NaOH for 45 minutes, washed with water, and stained in 0.1% aniline blue (dissolved in 0.1 M K_3_PO_4_) for 5 hours. Then the tissues were placed in a drop of glycerin on a slide and compressed under a cover slip. A fluorescence microscope (ZEISS scope. A1, Germany) with an excitation filter (BP: 395–440), a chromatic beam splitter (FT 460), and a barrier filter (LP 470) were used to examine the samples. A camera (CANON DS 126271, Japan) fitted to the microscope and the EOS Utility (Version 2.8.1.0, Japan) was used to make images. Lengths of the longest pollen tubes in each pistil were measured using Axio Vision LE (Axio Vs 40 V4.6.3.0, Germany). The times of the pollen tubes arriving at the ovary and penetrating the ovule were recorded.

### Onset of fruiting

In order to evaluate the pollinator’s effect on the reproductive success of greenhouse peach, the initial fruit set and the ovary growth from flowers pollinated by bees were recorded. We observed and compared the initial fruit set of Okubo rather than the final fruit set. This eliminates the need to consider other factors, such as resource competition, weather conditions, and horticultural management during the whole growing season. In each half-greenhouse, 3 peach trees were sampled for fruit-set in three years. At the balloon stage, flowers were labeled and counted to examine fruit set. Initial fruit set was monitored by counting fruit before the first commercial thinning. Initial fruit set was measured at the branch scale and a total of 30 branches were selected for each of the treatments.

In most peach flowers, ‘little fruit’ inflation occurred when petals and sepals wilted. To study ovary development in peach flowers pollinated by the different bee species, whole pistils were observed and photographed under a stereomicroscope. For the measurement of the ovaries, 12 pistils from each treatment were collected every other day, from pollination up to 15 days. A stereomicroscope (Olympus SZX16, Japan), together with a CCD camera (Olympus DP 71, Japan), was used to capture digital images of the pistils. The diameter of the ovary was measured using Axio Vision LE (Axio Vs 40 V4.6.3.0, Germany).

### Data analysis

A one-way ANOVA model was used to analyse the initial fruit set from flowers under different pollination treatments and Fisher’s LSD test was used to compare the mean of one treatment with the mean of the other. Stigmatic pollen loads are not normally distributed, so non-parametric Mann-Whitney *U*-tests were used to compare the effect of the different bee treatments on pollen deposition. The effects of bee species, year and sample plot on pollen-tube growth were assessed using GLM. Mean values of pollen-tube length in flowers pollinated by different bees were compared using a group *t*-test. Two-tailed probabilities for Fisher’s Exact Test were used to compare the percentages of pollen tubes in the ovary and the ovule at different time after pollinated by different bee species. Mean values for initial fruit set and ovary diameter from flowers pollinated by the different bees were compared using the group *t*-test. Statistical analysis were carried out in SAS 9.1.

## Results

### Peach mating system

**Fig 2:**
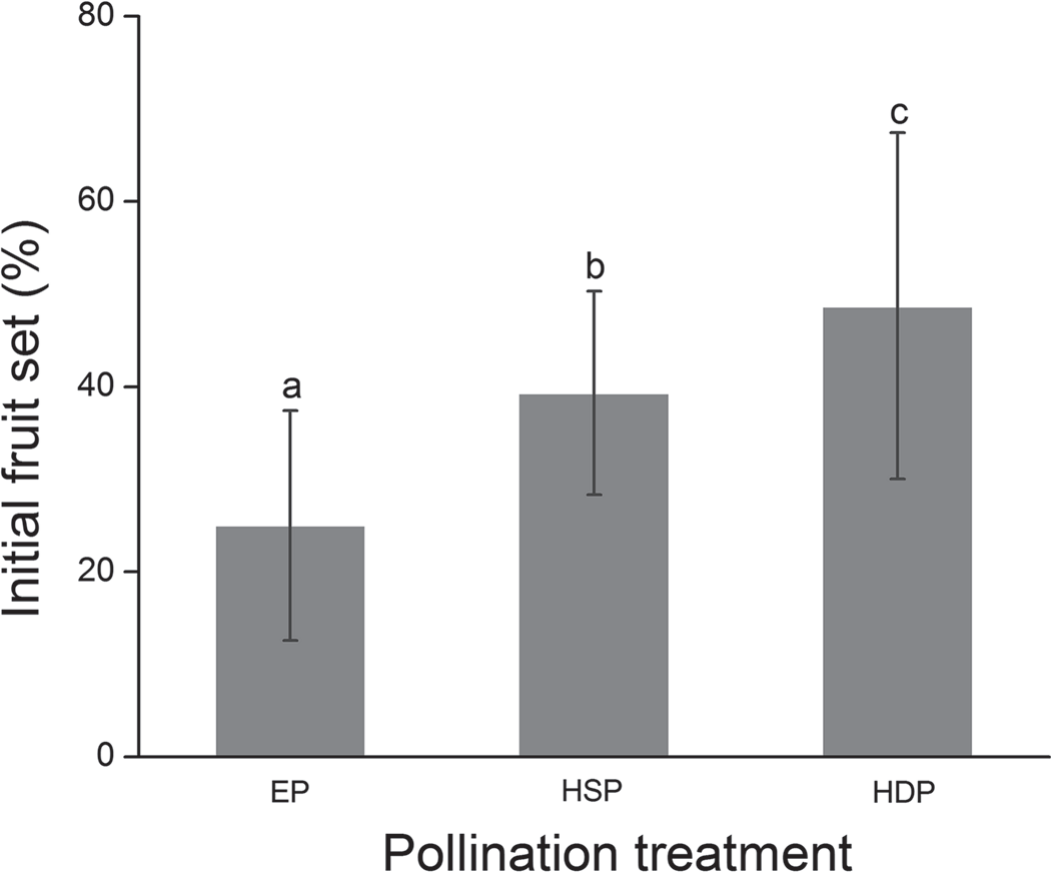
Initial fruit set of peach under greenhouse conditions. The initial fruit set (Mean ± SD) from flowers under different pollination treatments. Different letters indicate significant differences (*P* < 0.05) among pollination treatments using the Fisher test. EP: Pollinator exclusion; HSP: Hand-pollination with pollen from the same flower; HDP: Hand-pollination with pollen from a different flower.

We found that flowers from all pollination treatments produced fruit, although the initial fruit set of Okubo peach was significantly affected by the pollination treatment (*F*_2,68_ = 15.70, *P*<0.0001, Fig. 2). Flowers pollinated with pollen from different flowers had higher initial fruit set than self-pollinated flowers (*t*_45_ = 2.32, *P* = 0.02). Although flowers with pollinators excluded did produce fruit, the initial fruit set was much lower than that when flowers were hand-pollinated with pollen from the same flower (*t*_45_ = −3.26, *P* = 0.001) or with pollen from different flowers (*t*_45_ = −5.58, *P*< 0.001). We also monitored the development of the fruit from the pollinator-excluded flowers and found that these fruit grew more slowly than those from the hand-pollinated flowers. These results show that although autogamy dominates in Okubo, nonetheless pollinators are advantageous for peach in greenhouses.

### Pollen deposition

**Fig 3:**
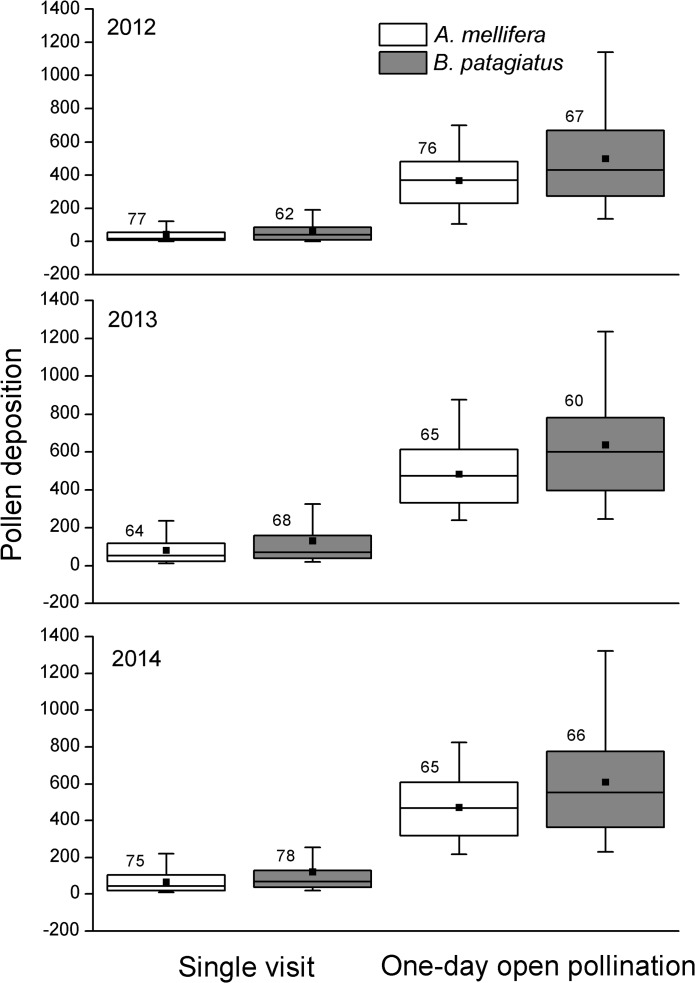
The number of pollen grains deposited on peach stigmas during single visits and one day of open pollination by *A*. *mellifera* and *B*. *patagiatus*. The bottom and top edges of the rectangle are the 25th and 75th percentiles, the horizontal line through the rectangle is the median, the solid square is the mean, and the tips of the whiskers indicate the fifth and 95th percentiles. The numerals on the boxes are the numbers of flowers sampled (Mann-Whitney *U* = 1774; *P* = 0.00943; n = 77, 62 for *A*. *mellifera* and *B*. *patagiatus*, respectively; medians: *A*. *mellifera* = 16, *B*. *patagiatus* = 39 in 2012. *U* = 2673.5; *P* = 0.02362; n = 64, 68 for *A*. *mellifera* and *B*. *patagiatus*, respectively; medians: *A*. *mellifera* = 51, *B*. *patagiatus* = 71 in 2013. *U* = 2126; *P* = 0.00337; n = 75, 78 for *A*. *mellifera* and *B*. *patagiatus*, respectively; medians: *A*. *mellifera* = 44, *B*. *patagiatus* = 67 in 2014) (*U* = 2043.5; n = 76, 67 for *A*. *mellifera* and *B*. *patagiatus*, respectively; *P* = 0.04227; medians: *A*. *mellifera* = 367.5, *B*. *patagiatus* = 430 in 2012. *U* = 2591; *P* = 0.00155; n = 65, 60 for *A*. *mellifera* and *B*. *patagiatus*, respectively; medians: *A*. *mellifera* = 472, *B*. *patagiatus* = 599 in 2013. *U* = 1586.5; *P* = 0.00985; n = 65, 66 for *A*. *mellifera* and *B*. *patagiatus*, respectively; medians: *A*. *mellifera* = 467, *B*. *patagiatus* = 554 in 2014).

We carefully monitored the foraging behaviour of the two bee species. Although *A*. *mellifera* has an advantage over *B*. *patagiatus* in the numbers of available individuals, *B*. *patagiatus* prefers to visit newly opened flowers more than *A*. *mellifera*, which makes it not difficult to get almost the same number of samples pollinated by *B*. *patagiatus* as pollinated by *A*. *mellifera*. Over the course of three years, data from a total sample of 216,208 peach stigmas visited once by *A*. *mellifera* or *B*. *patagiatus*, and a total sample of 206,193 peach stigmas visited for one day by *A*. *mellifera* and *B*. *patagiatus* were collected. *Bombus patagiatus* deposited more pollen grains on Okubo stigmas than *A*. *mellifera*, both during a single visit and during one day of open pollination (Fig. 3). Therefore *B*. *patagiatus* performed better than *A*. *mellifera* in pollen deposition efficiency when visiting Okubo flowers.

### Pollen-tube performance

**Fig 4:**
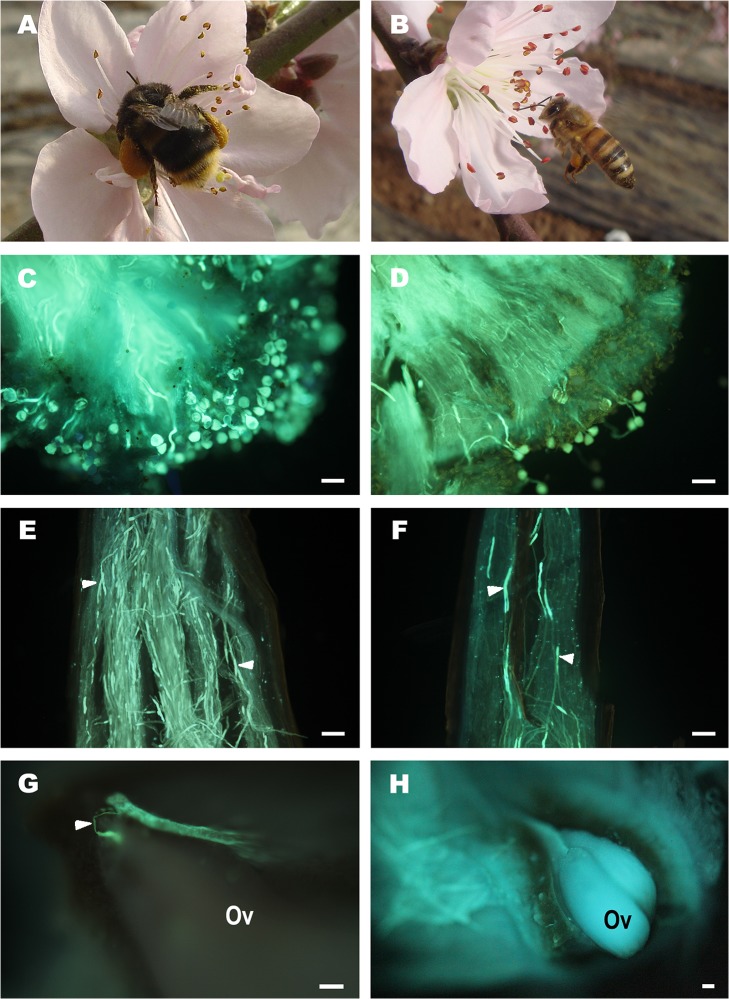
Pollen germination and pollen tube growth in peach flowers pollinated by *A*. *mellifera* and *B*. *patagiatus* under greenhouse conditions. Peach flower visited by (A) *B*. *patagiatus* and (B) *A*. *mellifera*. Pollen grain germination on peach stigma of flower pollinated by (C) *B*. *patagiatus* and (D) *A*. *mellifera*. Pollen tubes (white arrowheads) growth in style of peach flower pollinated by (E) *B*. *patagiatus* and (F) *A*. *mellifera*. (G) pollen tube (white arrowhead) penetrating ovule of peach flower 7 days after pollination by *B*. *patagiatus*. (H) ovule of peach flower 7 days after pollination by *A*. *mellifera*. All scale bars = 50 μm.

Our results show that sample plots from different parts of the greenhouse do not have a significant effect on pollen-tube growth, but bee species and year do have significant effects on pollen-tube performance (Table 1). In order to compare the effect of bee species on pollen-tube performance, the pollen-tube growth data from different sample plots in the same pollination treatment were combined within each year. Clear differences were observed between flowers pollinated by the two bee species on pollen-grain germination (Fig. 4C, D). A higher number of pollen grains germinated on the stigma of flowers visited by *B*. *patagiatus*, which appears to be the result of higher numbers of pollen grains deposited by these bees.

**Table 1:** General linear model of the effects of bee species, sample plot and year on pollen-tube growth along the style at each time after pollination of peach under greenhouse conditions.

Source	Day after pollination															
	1				3				5				7			
	d.f.	MS	*F*	*P*	d.f.	MS	*F*	*P*	d.f.	MS	*F*	*P*	d.f.	MS	*F*	*P*
Bee species	1	24.83	130.95	<0.0001	1	38.74	26.69	<0.0001	1	57.37	107.13	<0.0001	1	27.34	33.42	<0.0001
Plot	2	0.21	1.12	0.3356	2	0.05	0.04	0.9599	2	0.81	1.51	0.2321	2	0.17	0.21	0.8122
Year	2	10.81	57.04	<0.0001	2	6.18	4.73	0.0140	2	2.69	5.02	0.0112	2	7.27	8.88	0.0006
Bee species × Plot	2	1.35	7.10	0.0024	2	2.26	1.73	0.1897	2	0.42	0.79	0.4596	2	0.83	1.01	0.3711
Bee species × Year	2	0.73	3.90	0.0287	2	0.39	0.30	0.7439	2	0.54	1.00	0.3768	2	0.39	0.47	0.6272
Year × Plot	4	0.17	0.92	0.4610	4	0.77	0.60	0.6670	4	0.88	1.63	0.1840	4	0.39	0.47	0.7547
Bee species × Year × Plot	4	0.22	1.18	0.3365	4	0.44	0.34	0.8513	4	0.07	0.14	0.9672	4	0.26	0.32	0.8635
Error	38	0.19			42	1.30			41	0.54			43	0.82		
Total	55				59				58				60			

d.f., degrees of freedom; MS, Mean Square; *F*, F-statistic calculated; *P*, the probability that the table value for *F* is greater than the calculated value.

**Fig 5:**
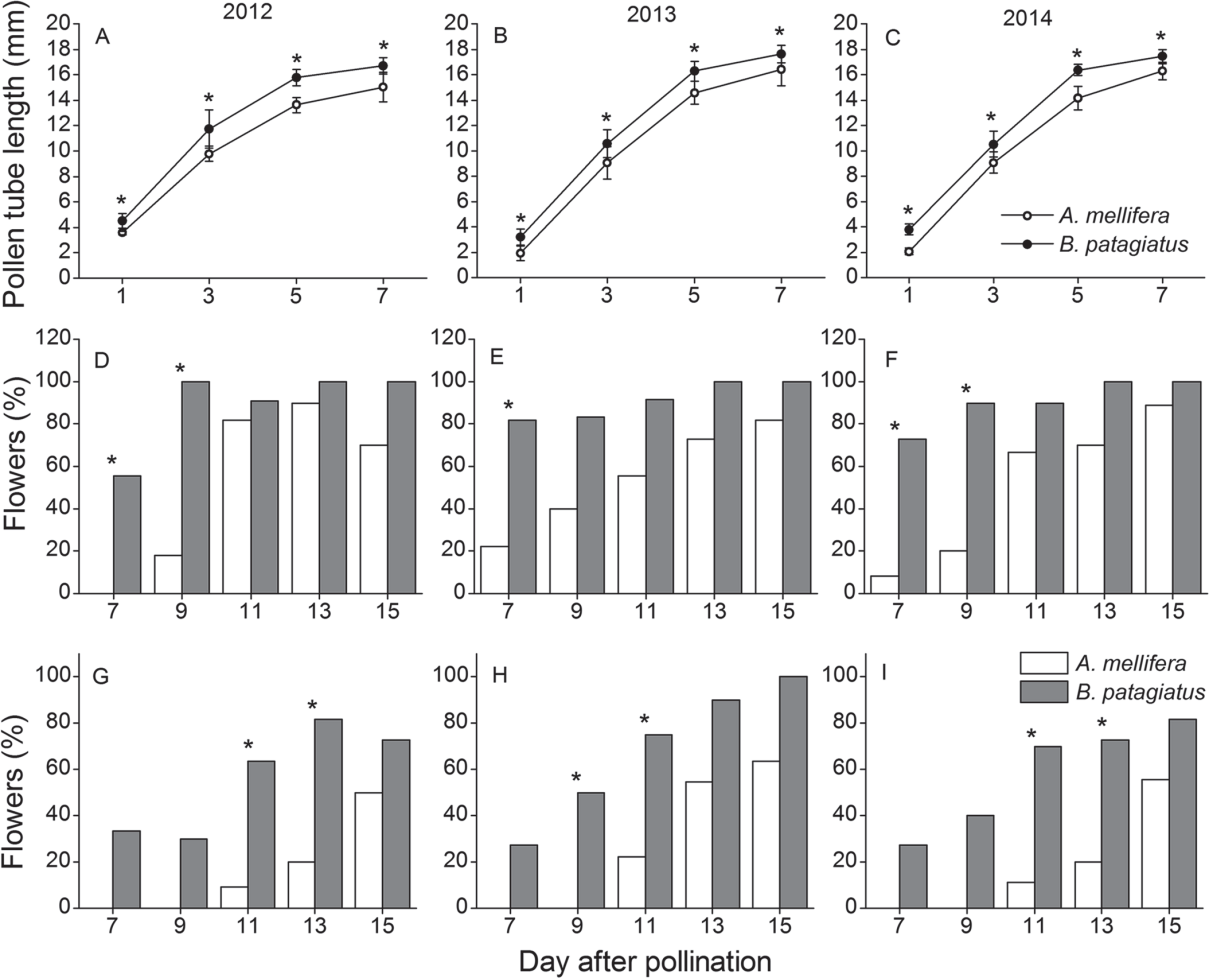
Pollen-tube performance in peach flowers pollinated by *A*. *mellifera* and *B*. *patagiatus* under greenhouse conditions. Lengths of pollen tubes (Mean ± SD) in the flowers after pollination at different times (1, 3, 5, 7 days) in year 2012 (A), 2013 (B), and 2014 (C). Percentages of investigated flowers with pollen tubes in the ovary after pollination at different times (7, 9, 11, 13, 15 days) in year 2012 (D), 2013 (E), and 2014 (F). Percentages of investigated flowers with fertilized ovules after pollination at different times (7, 9, 11, 13, 15 days) in year 2012 (G), 2013 (H), and 2014 (I). Within each year, flowers from all plots are combined here. Group *t*-test was used in graph A, B, C, and Fisher’s exact test was used in graph D, E, F, G, H, I. Single asterisks indicate significant difference at *P* < 0.05.

In our study, significant effects of bee species on pollen-tube growth along the peach style were recorded for each time period (1, 3, 5, 7 days) after pollination (Table 1, Fig. 4E, F). It took pollen tubes 7–11 days to arrive at the ovaries of flowers, depending on the different pollinator species. Within each year, flowers pollinated by *B*. *patagiatus* showed faster pollen-tube growth compared with flowers pollinated by *A*. *mellifera* within the first 7 days after pollination (S1 Table, Fig. 5A, B, C). More than half of the flowers pollinated by *B*. *patagiatus* were found with pollen tubes in the ovary 7 days after pollination. However, it took 11 days for pollen tubes to arrive at the ovary in half of the flowers pollinated by *A*. *mellifera* (Fig. 5D, E, F).

Beyond the differences between the two bee species in the time that pollen tubes took to arrive at the ovule, there was also an obvious stop to pollen-tube growth in flowers pollinated by *A*. *mellifera* (Fig. 4H). The pollen tubes in flowers pollinated by *B*. *patagiatus* penetrated the ovule as soon as they arrived at the ovary (Fig. 4G), so that half of the flowers were able to complete fertilization in 9 days (in 2013) or 11 days (in 2012 and 2014) after pollination. In flowers pollinated by *A*. *mellifera*, pollen tubes were first observed in the ovary 7 days (in 2013, 2014) or 9 days (in 2012) after pollination. But the pollen tubes did not penetrate the ovule until 2–4 days later. Half of these flowers completed fertilization within 13 days (in 2013) or 15 days (in 2012, 2014) after pollination, which is 4 days later than with *B*. *patagiatus* (Fig. 5G, H, I).

### Onset of fruiting

**Fig 6:**
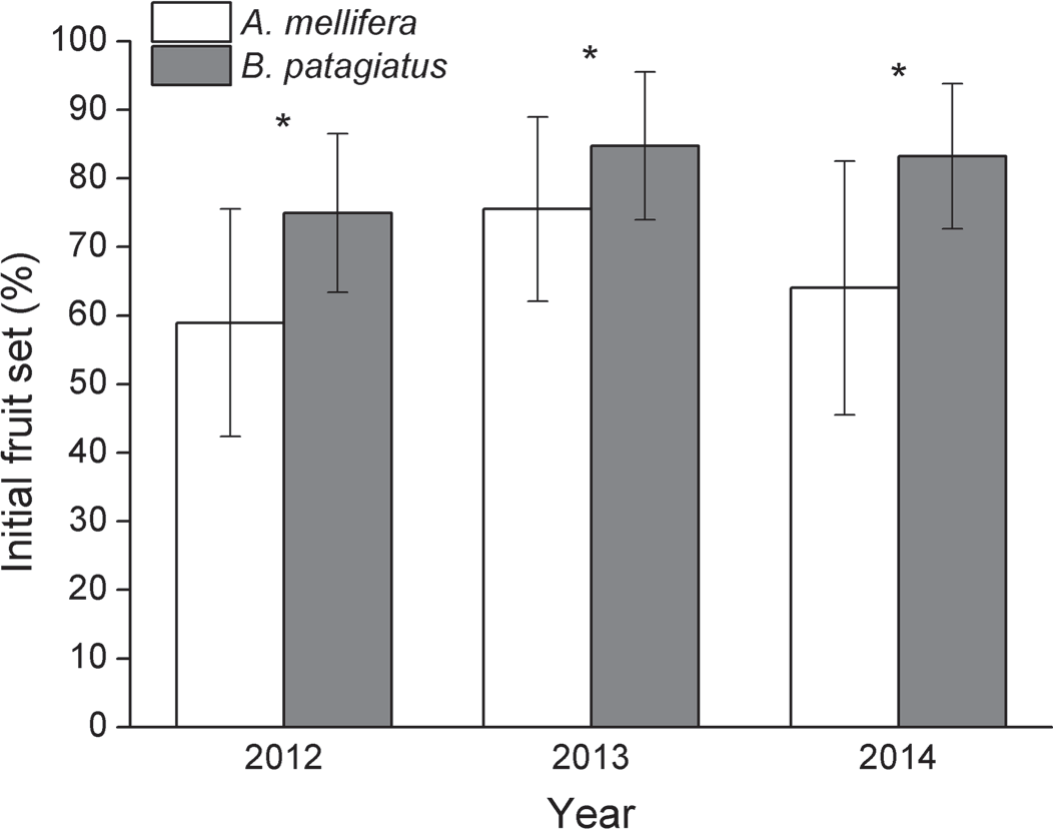
Initial fruit set of peach under greenhouse conditions. Initial fruit set (Mean ± SD) from flowers pollinated by *A*. *mellifera* and *B*. *patagiatus*. Within each year, branches from all plots are combined here. Single asterisks indicate significant difference at *P* < 0.05 by the group *t*-test.

We found that petal wilting, together with stigma browning, occurred 5–7 days after full blooming in Okubo flowers, and that the sepals dropped at the time of ‘little fruit’ inflation. Although *A*. *mellifera* colonies and *B*. *patagiatus* colonies were placed in greenhouses at same time, a difference in flower-drop was seen for flowers pollinated by the different bee species. In most flowers pollinated by *A*. *mellifera*, the flower dropped about 1–2 weeks after pollination. However, in most flowers pollinated by *B*. *patagiatus*, sepals and pistil did not drop until ‘little fruit’ developed. Fruit were counted as initial fruit set before first commercial thinning. In this study, we found that a significant difference in the initial fruit set: *B*. *patagiatus* (74.9±11.6%, 84.8±10.8%, 83.3±10.6% in 2012, 2013 and 2014, respectively) had a greater effect than *A*. *mellifera* (59.0±16.6%, 75.5±13.4%, 64.0±18.5% in 2012, 2013 and 2014, respectively) on initial fruit set in all three years (*t*_58_ = −4.33, *P*< 0.001; *t*_58_ = −2.94, *P* = 0.005; *t*_46_ = −4.94, *P*< 0.001 in 2012, 2013 and 2014, respectively, Fig. 6).

**Fig 7:**
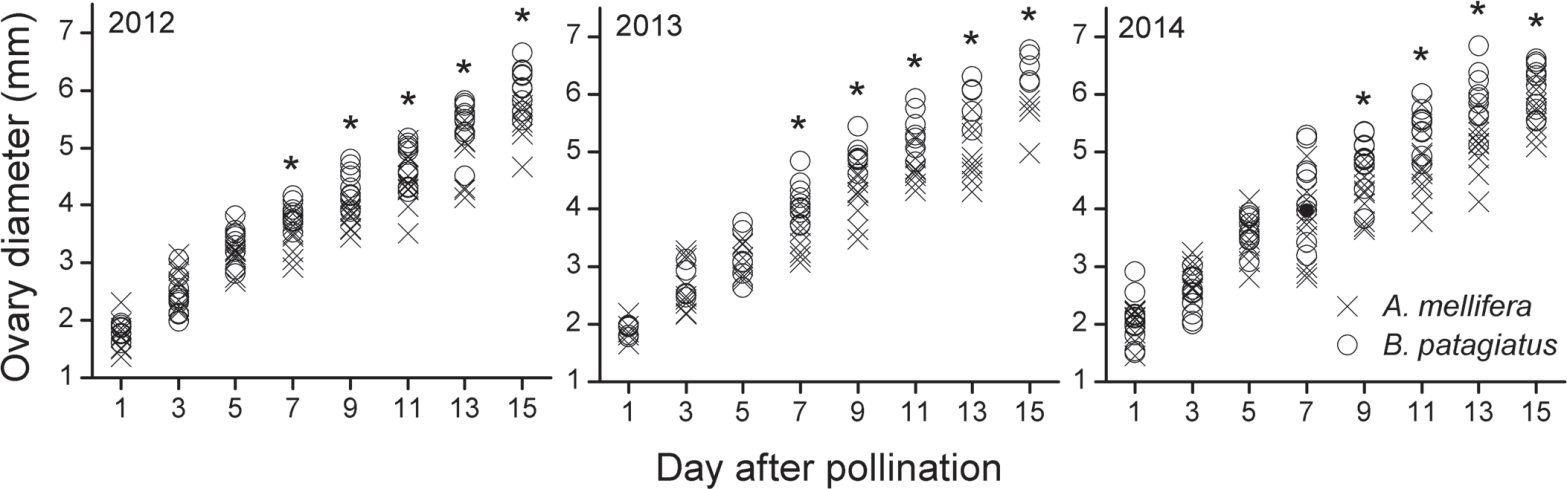
Early fruit development of peach under greenhouse conditions. Diameter of ovary, from pollination to 15 days later, in flowers by a single visit of *A*. *mellifera* and *B*. *patagiatus*. Single asterisks indicate significant difference at *P* < 0.05 by the group *t*-test.

The whole pistils were observed and photographed under stereomicroscope. The result show that there was no difference in ovary development within 5 days after visits by the different bee species (*t*_20_ = −1.45, *P* = 0.16; *t*_15_ = −0.91, *P* = 0.38; *t*_18_ = −0.90, *P* = 0.38 2012, 2013 and 2014, respectively, S2 Table, Fig. 7). However, 7 days after visits, ovary growth appeared faster in flowers visited by *B*. *patagiatus* compared with flowers visited by *A*. *mellifera* (*t*_19_ = −2.51, *P* = 0.011; *t*_17_ = −3.26, *P* = 0.005; *t*_19_ = −3.16, *P* = 0.005 2012, 2013 and 2014, respectively, S2 Table, Fig. 7).

## Discussion

Okubo peach is self-compatible and also partially self-fertile, as are most other peach cultivars [33]. This is confirmed by our study, in which even flowers that had pollinators excluded produced fruit. In Okubo, pollen grains have the strongest viability on the first day of full bloom, and then their viability declines gradually for 2–3 days [34]. During late blooming of Okubo, stamens become bent and the dehisced anthers drop pollen grains onto the stigma. We assumed that most of the bagged flowers had missed the best pollination period when ‘self’ pollen contacted the stigma [35, 36], or when deposition of pollen grains on the stigma is insufficient for successful pollination [6, 37, 38]. Neither the quantity nor quality of self pollen grains could meet full pollination requirements in most of the bagged flowers, which showed low fruit set. Therefore pollinators and timely pollination are essential for commercial production of peach under greenhouse conditions.

The number of pollen grains deposited on the stigma either per visit or per unit time have been suggested previously as predictors of relative pollination success [21, 22, 25, 39]. When visiting flowers for nectar or pollen, the body of the bee makes contact leaving pollen deposition on the stigma [40]. In our study, *B*. *patagiatus* deposited more pollen on the peach stigma than *A*. *mellifera* in both single visits and during one day of open pollination. Several factors may explain this difference. First, the larger size of *B*. *patagiatus* with more hair helps the bees make broader contact with the flower’s sex organs, helping to deposit more pollen grains on the stigma [41]. Second, when visiting peach flowers in greenhouses, bumblebees preferred to collect pollen, whereas honeybees preferred to collect nectar [29]. This may result in bumblebees making more pollen transfers among flowers than honeybees. Third, the floral display of Okubo peach encourages ‘side working’ by visiting bees. Honeybees often adopt side working on nectar-collecting visits. When side working, honeybees reach the nectaries without touching the stigma [17, 24, 42]. In our study, UV blocking polyethylene film was used in greenhouses by the owner. Compared with a normal polyethylene film layer, we found no effect of UV blocking film on honeybee or bumblebee behaviour. It appears that bee foraging behavior is unaffected by these UV conditions [43]. We also counted the pollen deposition after a day of open pollination, so as to combine single-visit deposition with the visit frequency, to predict pollination success on a larger scale [22, 44]. Our three years’ of results show that *B*. *patagiatus* has an advantage for pollen deposition both for single visit and for whole day of open pollination.

The period between pollination and fertilization is one of the most critical phases in the life cycle of flowering plants [36]. It has been reported that a distinct pollen-tube growth delay exists between pollination and fertilization in some angiosperms, such as in e.g. *Casuarina equisetifolia* (Casuarinaceae) [45], hazelnut *Corylus avellana* [46], *Corylus heterophylla* Fisch [47], and Fagales [48]. In almond, pollen tubes stop growing for 3 days at the base of the style before penetrating the ovule [49]. In peach, pollen tubes stop growing for 5 days [50–52]. These delays might be explained by immaturity of the pistil [53]. In some plants, the pistil matures from the stigma progressively downwards towards the ovary and ovule, so when pollen grains are deposited on the stigma, the base of the pistil and ovary are still immature. Larger numbers of pollen grains deposited on the stigma have been known previously to result in larger numbers of pollen tubes in the style, and greater competition among pollen tubes in the style promotes their growth rate [26, 54]. In our study, *B*. *patagiatus* showed an obvious increase in pollen deposition compared to *A*. *mellifera*, which was then combined with a faster pollen-tube growth within the style.

We report for the first time that a different interaction exists between pollen and pistil depending on the pollinator species and that this difference affects the crucial period between pollination and fertilization. In our study, the pollen tubes in peach flowers pollinated by *B*. *patagiatus* grew faster and penetrated the ovule as soon as they arrived at the ovary. In contrast, pollen tubes in flowers pollinated by *A*. *mellifera* grew more slowly and stopped for 2–4 days at the ovary before penetrating the ovule. These differences could be a consequence of the different pollen-deposition ability of the two bee species, because the number of pollen tubes affects the growth rate of the pollen tubes and determines the presence or absence of a growth stop. Pollen-pistil interactions are known generally to play an important role in regulating the pollen-tube growth in plants [50, 51, 53, 55]. In this study, the pollen tubes took 7 days to reach the base of the style. At the same time, significant difference occurred in ovary development 7 days after pollination by the two bee species. We suggest that more pollen grains on the stigma might not only result in a faster growth of the pollen tubes, but might also induce the maturation of the ovary and ovule in preparation for fertilization. The faster and continuous pollen-tube growth in the style allows the ovule to be fertilized earlier, and helps promotes faster ovary growth and higher initial fruit set. This brings an earlier fruit harvest and larger market profits in early summer.

## Conclusion

Our results show that although Okubo peach is self-compatible and capable of autonomous self-pollination, pollination by bees is nonetheless essential to securing commercial yields in greenhouses. We found that the native Chinese bumblebee, *B*. *patagiatus*, significantly increased the initial fruit set compared to the introduced western honeybee, *A*. *mellifera*. This difference is only partly explained by the difference in the amount of pollen deposited by the two bee species on the stigmas—there is also a different interaction between pollen and pistil depending on the pollinator species. Our results highlight the importance of (1) understanding the details of the pollination processes and (2) taking into account all the properties of a particular pollinator, especially indigenous bee species, when assessing their management cost.

## Supporting Information

S1 Dataset The raw data of the study. (RAR)

S1 Table Comparation of pollen tubes lengths, from pollination to days later, in flowers by a single visit of *A*. *mellifera* and *B*. *patagiatus* by group t-test. (DOCX)

S2 Table Comparation of ovary diameter, from pollination to 15 days later, in flowers by a single visit of *A*. *mellifera* and *B*. *patagiatus* by group *t*-test. (DOCX)
