# Supplementary material for: Managed Bumblebees Outperform Honeybees in Increasing Peach Fruit Set in China: Different Limiting Processes with Different Pollinators
Source: PLoS One. 2015 Mar 23;10(3):e0121143. doi: 10.1371/journal.pone.0121143 (PMC4370422; doi:10.1371/journal.pone.0121143)
Supplement: S1 Table — (DOCX) [file pone.0121143.s002.docx]

**S1 Table Comparation of pollen tubes lengths, from pollination to days later, in flowers by a single visit of *A. mellifera* and *B. patagiatus* by group *t*-test.**

| Day after  pollination | 2012 | | | 2013 | | | 2014 | | |
| --- | --- | --- | --- | --- | --- | --- | --- | --- | --- |
|  | t Statistic | DF | Prob>\|t\| | t Statistic | DF | Prob>\|t\| | t Statistic | DF | Prob>\|t\| |
| 1 | -4.65951 | 8.99 | 0.00119 | -4.70899 | 18 | 1.74968e-4 | -10.06633 | 16 | 2.50526E-8 |
| 3 | -3.9987 | 13.40 | 0.00143 | -2.75701 | 17 | 0.01347 | -3.45048 | 18 | 0.00285 |
| 5 | -7.64657 | 18 | 4.63571E-7 | -4.40566 | 17 | 3.86458E-4 | -6.71934 | 12.58 | 1.68707e-5 |
| 7 | -3.81648 | 16 | 0.00152 | -2.75629 | 18 | 0.013 | -3.45048 | 18 | 0.00285 |
