# Supplementary material for: Managed Bumblebees Outperform Honeybees in Increasing Peach Fruit Set in China: Different Limiting Processes with Different Pollinators
Source: PLoS One. 2015 Mar 23;10(3):e0121143. doi: 10.1371/journal.pone.0121143 (PMC4370422; doi:10.1371/journal.pone.0121143)
Supplement: S2 Table — (DOCX) [file pone.0121143.s003.docx]

**S2 Table C****omparation of ovary diameter, from pollination to 15 days later, in flowers by a single visit of *A. mellifera* and *B. patagiatus* by group *t*-test.**

| Day after  pollination | 2012 | | | 2013 | | | 2014 | | |
| --- | --- | --- | --- | --- | --- | --- | --- | --- | --- |
|  | t Statistic | DF | Prob>\|t\| | t Statistic | DF | Prob>\|t\| | t Statistic | DF | Prob>\|t\| |
| 1 | -0.7912 | 10.56 | 0.44624 | -0.14108 | 9 | 0.89091 | -0.45872 | 20 | 0.65138 |
| 3 | 0.93082 | 19 | 0.36362 | -0.34245 | 13 | 0.73748 | 1.03788 | 20 | 0.31171 |
| 5 | -1.45424 | 20 | 0.16139 | -0.90679 | 15 | 0.37885 | -0.90445 | 18 | 0.37770 |
| 7 | -2.50561 | 19 | 0.01074 | -3.26283 | 17 | 0.00458 | -1.18922 | 19 | 0.24900 |
| 9 | -4.12178 | 19 | 5.80159E-4 | -3.93342 | 15 | 0.00133 | -3.15741 | 19 | 0.00519 |
| 11 | -1.50669 | 19 | 0.00483 | -3.47024 | 15 | 0.00343 | -3.40068 | 19 | 0.003 |
| 13 | -3.02743 | 17 | 0.0076 | -3.96616 | 12 | 0.00187 | -2.90242 | 20 | 0.00881 |
| 15 | -3.71861 | 16 | 0.00187 | -3.97382 | 8 | 0.0041 | -2.31786 | 19 | 0.03177 |
